# The gendered socioeconomic impact of the COVID-19 pandemic on women with fistula repair in Uganda

**DOI:** 10.1525/agh.2023.1825767

**Published:** 2023-05-09

**Authors:** Mekaleya Tilahun, Mariko Costantini, Hadija Nalubwama, Justus K. Barageine, Florence Nalubega, Andrew Muleledhu, Alison M. El Ayadi

**Affiliations:** 1 School of Medicine, University of California San Francisco, San Francisco, CA, USA; 2 University of California, Berkeley, Berkeley, CA, USA; 3 Department of Obstetrics and Gynaecology, Makerere University College of Health Sciences, Kampala, Uganda; 4 Kitovu Hospital, Masaka, Uganda; 5 Kamuli Mission Hospital, Kamuli, Uganda; 6 Department of Obstetrics, Gynecology and Reproductive Sciences, University of California San Francisco, San Francisco, CA, USA; 7 Department of Epidemiology and Biostatistics, University of California San Francisco, San Francisco, CA, USA

**Keywords:** COVID-19, Female genital fistula, Vulnerable population, Uganda, Gender, Health service access, Economic impact

## Abstract

Public health mitigation strategies for SARS-COV-2 are effective in limiting the spread of COVID-19; however, these restrictions can create gendered socioeconomic impacts and further isolate marginalized communities from essential resources. Our qualitative study aimed to understand the gendered effects of the COVID-19 lockdown on a population of women in Uganda with a history of prior obstetric fistula repair, a group experiencing greater vulnerability and less support due to intersecting stigmatized identities. We conducted in-depth interviews among 30 women with prior genital fistula and corrective fistula repair surgery at 3 hospitals in Southern Uganda. We found that COVID-19 lockdown measures caused both financial and health-related impacts in this community such as inaccessibility of healthcare and loss of income. Understanding these experiences should inform strategies to ensure equitable, future pandemic responses.

## Introduction

Public health measures restricting mobility are effective in limiting the spread of SARS-CoV-2; however, these restrictions pose a threat to marginalized communities including children, the elderly, refugees, and women [1, 2]. After prior infectious-disease outbreaks such as Ebola and Zika, women experienced greater socioeconomic disadvantage than men. For example, during the Ebola outbreak in West Africa, food markets were closed during government-mandated quarantines, which upended the livelihoods of traders working in that sector, many of whom were women [3]. The global response to COVID-19 has taken the form of large-scale government mandates; however, the effects of this response may vary in severity within different communities. For many entrepreneurs in Uganda, businesses had to remain closed during the lockdown. These closures may have caused unintended downstream financial and socioeconomic consequences on groups depending on these businesses, such as women, children, and other marginalized groups [4].

Given the high level of female entrepreneurship in Uganda and their societal role as primary caregivers in the household, women in particular have been disproportionately affected by lockdown measures [4, 5]. Populations experiencing greater vulnerability and less support due to intersecting stigmatized identities, such as women with genital fistula, may be at an even greater disadvantage, and understanding the experiences and concerns of sub-populations can inform strategies to ensure equitable, future pandemic responses. Most women experiencing fistula are socioeconomically disadvantaged at the time of fistula development, and even after successful fistula repair, women continue to experience ongoing structural and economic disadvantages including familial abandonment and financial exploitation and abuse, in addition to physical ailments during their recovery [6]. The devastating socioeconomic consequences of the COVID-19 pandemic further compound onto their experiences; therefore, our research seeks to better understand the gendered impact of COVID-19 in Uganda on health and socioeconomic status among a cohort of women with prior genital fistula, a marginalizing experience with biopsychosocial impacts.

## Methods

We conducted in-depth interviews among 30 women with prior genital fistula and corrective fistula repair surgery at 3 hospitals in south, central, and eastern Uganda: Mulago Hospital (Kampala), Kamuli Mission Hospital, and Kitovu Hospital (Masaka). These facilities were selected for the volume of fistula repairs, geographic proximity, and prior research or clinical collaboration. At these facilities, fistula repair is provided as ongoing urogynecological services and is supplemented by several annual 2-week-long fistula repair camps, where human resources are concentrated to conduct a larger number of fistula repairs during one targeted time period. Our analysis was nested within a larger explanatory sequential mixed-methods study that sought to understand pregnancy experiences and outcomes following genital fistula repair. Quantitative data collection began in December 2019 but was on hold from March through September 2020 due to COVID-19 pandemic-related mitigation measures, which included a hold on all research activities. Due to the timing of our research and the potential for greater long-term pandemic impacts among this population, we sought to understand the early impact of the COVID-19 pandemic.

Women were purposively selected for interviews after having participated in the quantitative study phase. Quantitative participants were recruited from Mulago Specialized Women and Neonatal Hospital, Kamuli Mission Hospital, and Kitovu Mission Hospital. Over the period 2010–2015, approximately 3,000 women had fistula surgery at the targeted facilities [5]. Qualitative participants were purposively selected for diversity in postrepair pregnancy outcome (i.e., livebirth, stillbirth, spontaneous, or induced abortion) and mode of delivery for live and still-births (i.e., elective cesarean, emergency cesarean, and vaginal) in alignment with the objectives of our broader study.

Women were invited to participate in the qualitative study phase by a study team member. In-depth interviews were held between September 2020 and March 2021 in a private location and followed local COVID-19 protocols for research. Our interviews capture all lockdown-related experiences prior to March 2021. The Ugandan COVID-19 response underwent various stages of lockdown beginning March 2020 through June 2020. In March 2020, schools were closed, public transport was banned, and a nationwide curfew was implemented. Starting in May 2020, many restrictions eased with public transport resuming at reduced capacity and merchandise shops reopening with social distancing [7]. In-depth interviews were conducted by an experienced qualitative interviewer (HN) in a private room at a local healthcare facility. Interviews were conducted in the participant’s preferred language and lasted 1–2 h. Participants were paid 50,000 Ugandan Shillings ($13.37 USD) for research participation and travel. Interviews were audio-recorded with participant permission, transcribed verbatim, and translated into English for analysis.

Our in-depth interview guide sought to understand women’s concerns about the COVID-19 pandemic, the household impact of the COVID-19 pandemic, and associated mitigation measures across financial, food security, and mental health domains. We then asked women how the COVID-19 pandemic and associated mitigation measures impacted access, use, and receipt of health services across reproductive and maternal healthcare, child health services, infectious diseases, other incident conditions, and chronic conditions. Finally, we sought to understand how women and their families coped with these impacts.

Qualitative data analysis followed a two-stage systematic process rooted in phenomenological theory. The first stage involved data coding and classification by reviewing the transcripts for potential conceptual categories, using the in-depth interview guide questions as initial categories. Deductive codes that represent expected influences on the outcomes were applied to the data, taken from the existing literature and the interview guide. Next, inductive codes that emerged organically from the data were applied, representing themes unexpected by the researchers. In-depth interview transcripts were coded in Dedoose cloud-based software by a team of researchers including the Ugandan in-depth interviewer (HN), an American mixed-methods researcher (AE), an American medical student (MT), and an American undergraduate student (MC). Building on the in-depth interview guide, the team created a working codebook, which contained codes, their inclusion and exclusion criteria, and examples. The team jointly coded the first 4 transcripts to ensure standardized application of codes and resolved all discrepancies through discussion. The remaining 26 transcripts were assigned across the team for individual coding. The team met weekly to review coding progress and discuss questions. Coded data were thematically analyzed to describe the different dimensions and commonalities of each theme, their distribution across sociodemographic variables, and the patterns and linkages between themes.

### Ethical approval

Study procedures were reviewed and approved by the University of California San Francisco Institutional Review Board (IRB# 19-27901), the Mulago Hospital Research and Ethics Committee (MHREC# 1674), and Uganda National Council for Science and Technology (HS 2706). All participants provided written confirmation of informed consent.

## Results

Our results demonstrated a significant impact of the COVID-19 pandemic among study participants directly and indirectly across health, financial, food security, and mental health domains. Within each of these domains lie a subset of themes, which include the causes and consequences of participants’ experiences as well as the coping mechanisms they utilized in response to their situation. Participant demographic data are demonstrated in Table 1.

### Direct impacts of the COVID-19 pandemic

Participants and their families experienced varied levels of direct, physiological health impacts related to the COVID-19 pandemic. While participants did not disclose COVID-19 infection, they described respiratory disease morbidity occurring within their households, particularly among children:

*[The children] all fell sick that every week we were treating a different person. Malaria, flu, and cough. You know how children cough like they are coughing blood. I think maybe it’s because they sleep together, but every time one of them was getting better, the other fell sick. You know how villages are, we used to brew every herb we were directed to, and the children got better each day*. (Female participant 7, age 44)

To prevent and cope with these physiological health impacts, respondents described limiting movement and adopting COVID-19 mitigation measures, such as limiting visitors, washing hands, and wearing masks.

*We were not used to washing hands all the time, but we later got used to doing it. We were not used to the mask, and we would suffocate a few minutes after wearing it but since we want to live, we had to endure*. (Female participant 15, age 27)

### Indirect impacts of the COVID-19 pandemic

#### Healthcare access

Access to needed healthcare was significantly reduced among our study population and their families due to the ongoing COVID-19 pandemic, largely through government-implemented pandemic mitigation strategies, such as transportation bans, increased transport costs due to bans, reduced passenger numbers allowed on public transport, and reduced ambulance availability. Limited access to healthcare impacted participants and their family members in several ways including delays in access to ongoing or preventative healthcare services and medications.

##### Lack of provider availability

Participants described lack of access to healthcare providers for preventative care, such as vaccination and antenatal care, and specialized care for chronic illnesses, as appointments were limited or unavailable. Participants shared the following experiences:

*We couldn’t go to the hospital and the health workers that used to immunize in the community never came. So, when they eased transportation, we took [our child] to be immunized. They gave him the missed shots*. (Female participant 5, age 33)

*She wanted to go for a [antenatal] scan but she failed. However, God was merciful enough that she had a normal delivery despite not going for a scan at all*. (Female participant 30, age 40)

To cope with the lack of preventative care services, participants adopted a variety of strategies, including bypassing lockdown measures, postponing services, and purchasing medications in bulk. Examples of these experiences are as follows:

*[We did experience a situation where we had to take our baby for immunization but couldn’t go to the hospital.] We first gave it time because people weren’t allowed to move and weren’t allowed to gather. We took the child [for vaccination] after the lockdown was lifted*. (Female participant 19, age 27)

*My elder sibling had to get ARVs every month. He falls sick a lot. I called him and asked how he managing and he told me that he got the medication in bulk. He stays in Entebbe and gets his medication from Entebbe Grade B hospital*. (Female participant 22, age 28)

One participant shared that even though some pharmacies were closed during the lockdown, they discreetly bypassed lockdown measures to get their medication:

*They had closed the hospitals [due to COVID] and pharmacies weren’t allowed to operate as well. We had to go in hiding [to the pharmacy to get medication]*. (Female participant 21, age 26)

##### Increased medical care costs

Many medical services became more expensive during the COVID-19 lockdown, including hospital fees and medication prices. Some participants were unaffected by the increased medication costs. However, many were not able to purchase medications that they needed or only purchased the amount that they could afford.

Another described the effects of the rising costs of contraceptives, which resulted in some community members becoming pregnant due to their inability to afford it:

*[Contraceptives] are free at the government hospital but we have health workers in the village that administer them at 3,000shs ($0.80 USD). Before COVID-19 they were 2,000shs ($0.53 USD). Before they eased the lockdown, it was at 5,000shs ($1.34 USD) … You don’t get it. You will have to do everything possible to make sure that you get the money to get the injection if you don’t want to get pregnant … There are people who gave up on it and even got pregnant*. (Female participant 7, age 44)

To ease the burden of increased costs of medications and hospital visits, respondents shared the various strategies they used to utilize these resources including paying in installments or negotiating costs, accessing care at different locations (e.g., public vs. private facilities), using alternative treatments, foregoing other needed items, even food, or simply foregoing needed care altogether.

*The health worker who stays close to my home is patient. We go there for treatment and we pay in installments. My husband is understanding; when he knows that I need to go to the hospital, he finds ways of paying the bills slowly*. (Female participant 7, age 44)

*COVID affected everything. We now go to government hospitals because we don’t have money for private hospitals. They provide drugs but when they don’t have them then they prescribe and you buy it [elsewhere]. [Drug prices] have increased. If you need injectable or saline the prices are higher than imagined*. (Female participant 28, age 40)

*[My child and I] both fell sick [with malaria] but we didn’t have money. We would go to the hospital but they didn’t have malaria tablets and I wasn’t able to buy what they prescribed. We would buy Panadol whenever I earned like 1,000sh ($0.27 USD). I used to brew Omululuza to give to [my child]*. (Female participant 15, age 27)

Some participants described more extreme strategies to cope with the rising costs of hospital services and prescription medications. One respondent described foregoing food to afford medicine, and another considered sneaking their mother out of the hospital to avoid payment.

*My husband would always pay [for my prescription]. We would go without food but have the medicine because I would be in pain without taking the medicine.* (Female participant 13, age 41)

*Mother has been sick but I even thought of sneaking her out of the hospital because we didn’t have money. She was suffering from malaria and she also had a swollen leg. They charged us 90,000shs ($24.06 USD) for 3 days.* (Female participant 28, age 40)

##### Transportation challenges

To overcome transportation challenges, some participants described walking long distances on foot to access care, while others worked with drivers to take shortcuts by car or motorcycle. Others were unable to overcome these challenges, as described by one participant travelling by taxi:

*Currently, our problem is a means of transport. The taxi that I called in the morning went deep in the village to look for passengers then got stuck there. So, the car we used was too packed yet he charged us a lot. He even put us half way into the journey because it is not allowed to have that many people in a taxi. I spent hours at Kaliisizo because the taxi wanted to be filled and you can’t force someone to ride it.* (Female participant 11, age 33)

One participant discussed the consequences of postponing care for her mother’s illness due to increased costs of transportation to the hospital:

*My mother fell sick but we didn’t have money to take her to the hospital. By the time we got money to take her, they told us that she couldn’t be treated … She has cervical cancer. She is really not fine. What it changed is that we couldn’t go to the hospital for treatment. Before COVID, we had money to go to the hospital but now we don’t have any money to go.* (Female participant 7, age 44)

Consequences of breaching COVID-19 mitigation strategies could be severe, and many participants described harrowing individual, family, or community member experiences of being beaten by security personnel, even where transportation was rationalized, as shared within the following narratives:

My grandmother has illness like ulcers and hypertension [which required treatment]. One of her sons went to the RDC (Resident District Commissioner) and got the form to let them go to the hospital but they were beaten by police offices. You know they would beat people without analyzing the situation. While they were beating my uncle, they saw an older person behind the boda waving the approval form, that’s when they let them go …

*Transport in the villages is hard and we were not allowed to carry people on a boda. There was also a woman who was beaten because she was seated on a boda and she lost her pregnancy. No one knows if those police officers were arrested but the woman died. They didn’t mind about that [her pregnancy]; they would just beat if they saw you seated on a boda.* (Female participant 26, age 23)

*I have even walked [to the hospital] during pregnancy when we had no means of transport, in an emergency …On my way back, no boda rider was willing to take us back because they would be arrested or beaten up.* (Female participant 27, age 28)

Respondents reported a variety of coping strategies to manage the transportation restrictions in place, from postponing care, to using alternative modes of transportation, such as walking, that were accepted within the COVID mitigation protocols, to using delivery services for medications or purchasing them in bulk.

*Well, you had to start walking early in the morning to go to the hospital and when you got there, you had to make sure they handled you fast such that you return home on time … It is about 20 miles from our home to the town.* (Female participant 17, age 30)

*It has affected us. If a child falls sick at night, you can’t take her to the hospital and the ambulances don’t come at night. You have to wait until morning. At the peak of COVID-19, my child got a flu …We were all scared and didn’t sleep the whole night. I waited for whatever was going to happen but God was merciful; that morning, the child was feeling better and we took her to the hospital. We walked there because even bodas were not allowed to carry passengers.* (Female participant 10, age 27)

Participants reported substantial relief when transportation-related lockdown measures were lifted.

#### Financial consequences

The COVID-19 lockdown caused reduced, or complete loss, of income for many study participants, particularly those who had small businesses. Even after restrictions ended, the lockdown had ongoing impacts on household income.

*Covid treated us so badly; it has kept us in poverty. Before Covid came, I was employed. I used to fry potato chips at the roadside, but that was affected because we were just there and had nothing to do. We [resumed] work but business is not running so smoothly. We do not have enough capital.* (Female participant 4, age 32)

We were mostly worried about closing places of work but my partner wasn’t affected because he’s in the food sector. So, they still worked even though not as much but it was better than those who were sitting at home.

Participants receiving financial support from their partners described receiving less due to their decreased income compared to before COVID-19.

*[My partner] works and gives me the little that he makes and I use it sparingly. His finances reduced [compared to before COVID] and he is just doing whatever he can to make ends meet.* (Female participant 19, age 27)

*[The COVID situation] has affected me because business has been really slow. My husband supports just that his income was also affected. He is a builder.* (Female participant 25, age 28)

To cope with the impacts of unemployment and reduced income, respondents sought out other earning opportunities and negotiated ongoing financial commitments. One participant shared the difficulty she faced paying rent:

*I don’t work because I used up all my business capital during COVID. [Before COVID] I was cooking snacks. [I survive with the child by finding] some work like I told you. I can do some work and earn 2,000–3,000shs ($0.50–$0.80 USD). I also have friends in saloons who call me to plait cornrows and they pay me like 4,000shs ($0.80 USD). With that, I managed to survive and also pay the landlord. [When I first stopped working,] I told the landlord to let me be for a while. However, he once gave me a job and I was able to pay rent. He asked me to weed his groundnuts. If I didn’t do it, he would have chased me from the house*. (Female participant 15, age 27)

A farm owner shared how selling produce prior to harvest allowed them to secure funds:

*We farm and grow food because if I am lucky and finish planting on time by June I will be harvesting. Even if you haven’t sold them yet or they are not ready, you can get someone to buy the produce before harvest if they have the money. I don’t worry about it at all because I can sell something.* (Female participant 11, age 33)

#### Food insecurity

Participants reported various pandemic impacts on food security. For some, food security was a significant concern, whereas for others, it did not represent any additional stress. Individuals reporting less of an impact on food security were those who regularly cultivated crops for feeding their families or selling:

*For us people in the village, we don’t buy food and we don’t rent. I had cassava and matoke, and during COVID the harvests were good. I heard on the radio that people are dying due to lack of food. We had food and the children gained weight. I realized that my family was easy to manage because I could harvest matoke and eat it for a few days and I had cassava too. So, it wasn’t so bad compared to those in town who had to buy everything to eat and even pay rent. It’s not like I am fine, but it wasn’t so bad. Of course I was affected because children are not going to school and things are not moving, [but we had food].* (Female participant 11, age 33)

One participant who worked on a farm shared that people stole the produce that they planted just at the start of the lockdown. Many of their crops also were not harvested due to the lack of quality of the crops, which affected them significantly as well.

*It was very bad. It affected us a lot. COVID-19 got us after we had just planted our crops and we still lacking food a lot to this day. They used to steal the food from our gardens; they would harvest the cassava and also uproot the sweet potatoes at night. They would even cut down bananas when they were still young. [Those people were] thieves. I think they used to take them somewhere to sell them. We don’t really know. The beans dried out, maize didn’t bear fruits so, we have been in a very hard condition.* (Female participant 28, age 40)

Participants coped with food insecurity through reducing the quantity of food eaten, eating lower cost food, or foregoing preferred foods. Others accessed food through government and church sources.

*We get just a little food to eat; if we got a quarter of rice to cook, that will take us to the next day. The children would cry until they got tired. We would also take porridge during day.* (Female participant 28, age 40)

*We managed [to survive]. I am trying to tell you that it wasn’t easy to find something to eat. We would buy posho or even get food from the church. Sometimes my partner would find ways of buying us something to eat.* (Female participant 22, age 28)

Some workplaces sought to mitigate the decreased income:

*[Despite being impacted financially,] I didn’t have any problem [getting enough food] because I am provided with food at my workplace. [I continued working during the lockdown.]* (Female participant 21, age 26)

#### Mental health impact

The COVID-19 pandemic and lockdown impacted the mental health of respondents, all of whom had varying emotions ranging from fear to worry. Fear was expressed by many participants and was due to uncertainties about COVID-19 and its consequences on personal health, social status, and financial impacts of the lockdown. Many fears stemmed from beliefs of COVID-19 being deadly and uncurable.

*Personally, I thought that if my child was sick [with COVID], there is no way the rest of us were fine. So, when my child fell sick, I thought that we were going to die one by one …I was really scared because I knew that if I told anyone about it, no one would want to come close to me. Remember that I run a business, so I don’t think people would still come to my shop. Imagine if they found out about it! Do you think they would still come?* (Female participant 10, age 27)

*What worried me the most is the way it came and they said it does not heal and that there is no medicine and so you would get worried as you have to protect your children as well as yourselves*. (Female participant 12, age 27)

Other fears were related to COVID mitigation efforts, for example, one participant shared her fear of what she would do if she needed transportation and it wasn’t available:

*The bad things were maybe the fear of illnesses. Fear that in case a child falls sick, where do you take him when transportation wasn’t available?* (Female participant 23, age 29)

One participant described her fear about the indefinite nature of COVID-19 and how it would continue to impact their lives in the future:

*I wasn’t worried about getting the disease because we were not moving, but I was scared about how we were going to survive if it went on for a longer time.* (Female participant 22, age 28)

Participants also felt worried about various factors, including the direct effects of COVID-19 on the health of loved ones as well as having basic needs met during the lockdown. In addition, many participants were worried about their children’s education given the closure of schools.

*We would get worried in case you have a relative that stays far away from you as you would be wondering how they are doing and how they are surviving. Two of my children work from town, one is a conductor and the other works in a mattress and foam industry, I would always think about them. They did not fall sick but I was always worried about how they are feeding, how they manage rent*. (Female participant 18, age 44)

*Our child has now become stupid … If I call him to come and study, you can feel that he is getting hypertensive if you touched him (laughs) … His sister wants to study so, sometimes you can hear her telling him, “let’s study.” That’s when you hear him getting angry and he tells her, “let’s go for jackfruit.”* (Female participant 11, age 33)

The impact of the closure of schools and lack of education during this time go beyond academics; some respondents were worried that their children had a higher chance of becoming infected, given their increased freedom to socialize if they were not in school. For families whose children were not in school, having their children at home during the day added expenses and responsibilities that were not there prior to COVID-19.

*We lacked the basic needs. The children also had needs since they were now at home but we couldn’t provide for them. We had to struggle for everything. We were always worried. It has not been easy.* (Female participant 7, age 44)

*I was affected psychologically; I was always worrying for my children about what they are going to eat or what to do or how to go through that COVID situation.* (Female participant 29, age 23)

Participants were also worried about traveling during the COVID-19 lockdown due to increased costs of transportation and strict, oftentimes violent, enforcement of the lockdown curfew. One respondent shares their worry and fear of the police during lockdown if citizens are found outside of their homes after dark.

*Yes, you had to be worried because whenever you went anywhere, you had to make sure that you returned home early enough because if it went dark before you returned home, they would beat you up. They would beat you up if it went dark when you are still loitering around or if you are still in town. [The police] would beat you up.* (Female participant 17, age 30)

Participants shared their strategies to help cope with fears about contracting COVID-19; many felt better implementing preventative strategies such as washing hands and isolating to prevent COVID-19 infection.

## Discussion

Our research sought to understand the gendered socioeconomic and health impacts of the COVID-19 lockdown on women with prior fistula repair in Uganda–a vulnerable group due to the sociodemographic and contextual factors resulting in fistula development. Mitigation efforts affected physical and mental health as well as participants’ employment and income. Given their health status and socioeconomic position, this population requires a context-appropriate government approach to aid in supporting against harmful effects of the COVID-19 lockdown.

### Physical and mental health

Hospital and clinic inaccessibility during the COVID-19 pandemic caused several health consequences in our study population, including lack of access to preventative and emergency healthcare services. Those living in remote areas of Uganda where motorized transport is required to access care walked long distances to reach hospitals. For emergency care, including obstetric emergencies, mobility restrictions, and delays in intervention can lead to adverse maternal and perinatal outcomes [8]. For example, by April 2020, seven laboring Ugandan mothers and two babies died due to hospital inaccessibility and delayed obstetric care [9]. For laboring women in remote areas, lack of timely services can also lead to prolonged, obstructed labor and development of obstetric fistula. Data show that many times, delivery leading to fistula results in stillbirth [10]. Individuals in our study population may be more strongly impacted by delays in obstetric care, as their history of fistula repair puts them at greater risk of fistula recurrence. Further efforts to reestablish continuous, longitudinal care and improve access to providers are needed.

Participants experienced mental health effects due to the pandemic including feelings of fear and worry surrounding COVID-19 disease symptomatology and prognosis as well as ongoing financial effects of the lockdown. The lockdown caused many Ugandans to experience life-altering events, such as food insecurity, fragmented social relationships, unemployment, poverty, and increased violence by police [2]. Previous studies linked exposure to traumatic events with mental health impacts including trauma and stress-related disorders [11]; therefore, it will be important to understand the long-term mental health consequences in order to adequately provide mental health services in future pandemic responses.

The Ugandan government guidelines facilitating necessary transport during the pandemic advised individuals needing hospital care to seek prior permission from Resident District Commissioners [12]. Our study findings documenting violent responses to valid transportation needs are concerning, and indeed, while the lockdown lasted until early May [13, 14], by the end of July 2020, there were 12 reports of people who had allegedly been killed by security officers in acts of excessive force when enforcing lockdown measures [15]. The systems put into place by the government to protect citizens are advertently harming them. Governments should recognize that restrictive measures may deprive vulnerable groups from equitable access to healthcare and cause them to face extra challenges in accessing food and other basic needs [16]. As the pandemic continues, public health measures and COVID-19 mitigation strategies should be centered on a human-rights approach to protect vulnerable groups who are more severely impacted by restrictive lockdown measures.

### Employment, income, and food security

The effects of the COVID-19 lockdown on employment, income, and food security may further reduce the socioeconomic status of the women in our population. Given their history of genital fistula, a lower socioeconomic status may increase the risk of fistula recurrence and its consequences. Increased costs of healthcare services created a financial burden and healthcare disparity for women in the community. As a result of increased costs, some women were unable to purchase contraceptives, which may lead to unintended pregnancies. Especially given the limited access to emergency obstetric care in our population, unintended pregnancy can cause serious adverse maternal and perinatal consequences [10, 17]. To improve the socioeconomic status of women unable to access emergency care, future pandemic interventions should classify all preventative healthcare services, including contraceptive services, as essential and ensure equitable distribution and access through existing community-based clinics and pharmacies.

The COVID-19 lockdown also increased poverty in Uganda from 18% before the pandemic to 28% within the first half of 2021 [18]. Unemployment disproportionately impacted women, including those in our study, given the high level of female entrepreneurship in the food and agricultural sector. It is also important to consider women’s societal role as caretakers in Uganda. Because of this, their return to work after the pandemic will likely depend on the fulfillment of their other home responsibilities, further hindering financial independence and security. After food market closures during the Ebola outbreak in West Africa, less than 20% of female market workers returned to work 1 year after the first case was detected, in contrast to over 60% of males [3]. Moving forward, it will be important to monitor the long-term financial effects that business closures have on women in this population as well as implement strategies to allow women to return to work sooner.

Our results demonstrated that income losses led to increased food insecurity among farmers and nonfarmers alike. Participants working in agriculture were able to provide for themselves by utilizing the crops they grew; however, they harvested and consumed a limited variety of foods. In addition, they earned less income from crop sales due to low prices. Our findings aligned with another study exploring food insecurity in Uganda during the COVID-19 lockdown, which reported greater food insecurity among rural farming families given differing reliance on farm income, with urban farmers diversifying their income sources and rural farmers feeding their families [19].

The financial impact of the lockdown on our participants extended to children’s education, which has already experienced significant disruptions based on lengthy school closures, particularly in Uganda whose public schools were closed for nearly 2 years [20]. Due to reduced income, participants may be unable to afford school fees when they reopen, which will exacerbate educational disruptions for their children. Children who are out of school are at increased risk of exploitation, poverty, and educational disparities [21]. When schools were closed, the children of our study participants interacted with other peers in the community and remained idle during the days. Without the safety net of schools, increased sexual activity may lead to unwanted, and riskier, pregnancies due to lack of healthcare access. During the Ebola outbreak in Sierra Leone, teenage pregnancy increased by 65%. In Northern Uganda, there have even been reports of young women forced to sell sex for money, food, and sanitary products [22]. A similar increase in unwanted pregnancy among young women is possible in Uganda after the COVID-19 pandemic, and complex and multifactorial efforts in reproductive services, as well as reintegration into schools, will be necessary to address this issue.

### Strengths and limitations

Our study’s qualitative approach led to a nuanced understanding of a specific, vulnerable population. Our participants are considered to have low socioeconomic status, given their health factors related to developing fistula; therefore, it was expected that the COVID-19 pandemic and restrictions could potentially impact this population more severely.

The interviews were conducted in August 2020, after the most severe COVID lockdown in Uganda. As the pandemic was fairly new, our study focused mainly on the indirect effects of COVID-19 on participants’ health; however, future studies should consider the direct effects of COVID-19 on this population, as vulnerable populations may have higher COVID-19 rates and women of reproductive age who become pregnant may experience more severe outcomes than people who are not pregnant [23].

## Conclusions

The COVID-19 lockdown caused significant financial and health impacts for women in Uganda with prior obstetric fistula repair. These effects may cause lasting consequences for a community already grappling with marginalization and socioeconomic burden. Early lockdown measures, including shutting down public transportation and nonessential businesses, isolated vulnerable groups from essential healthcare services and increased poverty and food insecurity. Likewise, the inaccessibility of emergency maternal healthcare, educational disruptions for young children, and unemployment in the food sector, which is highly dominated by women, produced gendered impacts particularly affecting the most vulnerable communities. Future pandemic responses should adopt a human-rights-centered approach that ensures equitable distribution of resources to marginalized communities.

## Figures and Tables

**Table 1.** Demographic data of the study participants

**Age**	29.5 (27.0–33.0)
**Relationship status**	
Single, never married	2 (6.7%)
Married or domestic partnership	20 (66.7%)
Widowed	2 (6.7%)
Separated	6 (20.0%)
**Same partner/husband during fistula development**	
No	9 (45.0%)
Yes	11 (55.0%)
**Same partner/husband during fistula surgery**	
No	6 (30.0%)
Yes	14 (70.0%)
**Total lifetime schooling**	
None	2 (6.7%)
Some primary	9 (30.0%)
Completed primary	12 (40.0%)
Some secondary	7 (23.3%)
**Employment status in the past year**	
Informal employment for wages	7 (23.3%)
Self-employed	5 (16.7%)
Housewife	12 (40.0%)
Not employed	6 (20.0%)
**Personal total monthly income**	
None	10 (33.3%)
10,000 or less	2 (6.7%)
50,000–100,000	13 (43.3%)
100,000–500,000	5 (16.7%)
**Total monthly income of everyone residing in the home**	
None	1 (3.3%)
10,000 or less	1 (3.3%)
50,000–100,000	10 (33.3%)
100,000–500,000	16 (53.3%)
More than 500,000	2 (6.7%)

## Data Availability

The data that support the findings of this study are available from the corresponding author upon reasonable request.
